# Costs of seasonal influenza vaccination in South Africa

**DOI:** 10.1111/irv.12987

**Published:** 2022-03-30

**Authors:** Heather Fraser, Winfrida Tombe‐Mdewa, Ciaran Kohli‐Lynch, Karen Hofman, Stefano Tempia, Meredith McMorrow, Philipp Lambach, Wayne Ramkrishna, Cheryl Cohen, Raymond Hutubessy, Ijeoma Edoka

**Affiliations:** ^1^ SAMRC Centre for Health Economics and Decision Science—PRICELESS SA, School of Public Health, Faculty of Health Sciences University of the Witwatersrand Johannesburg South Africa; ^2^ Health Economics and Health Technology Assessment, Institute of Health and Wellbeing University of Glasgow Glasgow UK; ^3^ Influenza Division Centers for Disease Control and Prevention Atlanta Georgia USA; ^4^ Influenza Program Centers for Disease Control and Prevention Pretoria South Africa; ^5^ MassGenics Duluth Georgia USA; ^6^ School of Public Health, Faculty of Health Sciences University of the Witwatersrand Johannesburg South Africa; ^7^ US Public Health Service Rockville Maryland USA; ^8^ Department of Immunization, Vaccines and Biologicals World Health Organization Geneva Switzerland; ^9^ Communicable Disease Cluster National Department of Health Pretoria South Africa; ^10^ Centre for Respiratory Diseases and Meningitis National Institute for Communicable Diseases of the National Health Laboratory Service Johannesburg South Africa; ^11^ Health Economics and Epidemiology Research Office, Department of Internal Medicine, School of Clinical Medicine, Faculty of Health Sciences University of the Witwatersrand Johannesburg South Africa

**Keywords:** cost analysis, health care economics, influenza vaccine, preventive health programmes, South Africa

## Abstract

**Background:**

Influenza accounts for a substantial number of deaths and hospitalisations annually in South Africa. To address this disease burden, the South African National Department of Health introduced a trivalent inactivated influenza vaccination programme in 2010.

**Methods:**

We adapted and populated the WHO Seasonal Influenza Immunization Costing Tool (WHO SIICT) with country‐specific data to estimate the cost of the influenza vaccination programme in South Africa. Data were obtained through key‐informant interviews at different levels of the health system and through a review of existing secondary data sources. Costs were estimated from a public provider perspective and expressed in 2018 prices. We conducted scenario analyses to assess the impact of different levels of programme expansion and the use of quadrivalent vaccines on total programme costs.

**Results:**

Total financial and economic costs were estimated at approximately USD 2.93 million and USD 7.91 million, respectively, while financial and economic cost per person immunised was estimated at USD 3.29 and USD 8.88, respectively. Expanding the programme by 5% and 10% increased economic cost per person immunised to USD 9.36 and USD 9.52 in the two scenarios, respectively. Finally, replacing trivalent inactivated influenza vaccine (TIV) with quadrivalent vaccine increased financial and economic costs to USD 4.89 and USD 10.48 per person immunised, respectively.

**Conclusion:**

We adapted the WHO SIICT and provide estimates of the total costs of the seasonal influenza vaccination programme in South Africa. These estimates provide a basis for planning future programme expansion and may serve as inputs for cost‐effectiveness analyses of seasonal influenza vaccination programmes.

## 1 INTRODUCTION

Human (seasonal) influenza viruses are considered a global public health threat, causing substantial morbidity and mortality annually in low‐ and middle‐income countries (LMIC), with particularly high mortality rates estimated among vulnerable individuals in sub‐Saharan Africa.^1^ For example, prior to the COVID‐19 pandemic in South Africa, infection with influenza virus accounted for over 56,000 hospitalisations and 11,000 deaths annually,^2^ with a high economic burden of approximately $270 million.^3^ The health and economic burden of seasonal influenza is exacerbated by the high prevalence of comorbidities in South Africa, such as HIV, tuberculosis and other non‐communicable chronic diseases, which are associated with increased risk of severe influenza.^4^, ^5^

Influenza vaccination can prevent influenza‐associated illness, including among individuals at risk of developing severe complications of the disease.^6^, ^7^ The World Health Organization (WHO) recommends that individuals at risk of severe influenza‐associated illness be considered for vaccination as part of seasonal influenza vaccination programmes.^8^

In 2010, the South African National Department of Health (NDoH) introduced the trivalent inactivated influenza vaccine (TIV) into the public health system free of charge.^9^ Annually, the campaign targets selected individuals at risk of severe influenza‐associated illness for vaccination. As of 2017, this includes those aged ≥65 years, pregnant women, HIV‐infected individuals and individuals aged over 6 months with other underlying medical conditions (UMC), including tuberculosis, heart disease and lung disease.^9^, ^10^ While all individuals in these target groups are eligible for vaccination as part of the vaccination campaign, pregnant women and HIV‐infected adults are prioritised by the campaign.^9^ An overview of the influenza vaccination programme in South Africa can be found in the Supporting Information.

Few persons in targeted groups are vaccinated each year. For example, only 5% of the total number of doses required to cover the prioritised groups were procured for use in the public health sector in 2018 (approximately 900,000 doses).^11^, ^12^ This large gap in available doses may be explained by limited economic evidence, particularly in LMIC, to support the case for increased investment in seasonal influenza vaccination, in spite of growing evidence on the benefits of seasonal influenza vaccines in high‐risk groups.^13^, ^14^, ^15^ To close these gaps, the WHO has developed a standardised mechanism to collect both national and global influenza burden data; to better determine the economic burden among risk groups; and to determine the costs and cost‐effectiveness of influenza vaccination in low resource settings.^16^, ^17^ South Africa has used this mechanism to good effect in estimating the disease and economic burden of influenza as well as the cost‐effectiveness of the influenza vaccination programme.^2^, ^3^, ^18^ An assessment of programme delivery and vaccine procurement costs would be useful to identify opportunities for improved efficiency, as well as to plan for potential expansion of the seasonal influenza vaccination programme.^19^, ^20^ Furthermore, although a decline in confirmed influenza cases during the COVID‐19 pandemic has been reported globally (including in South Africa) due to public health measures, this may result in future compensatory increases in influenza infections.^21^ This makes it even more important to understand the costs of the influenza vaccination programme to ensure effective planning and preparedness for a potential influenza epidemic.

This study estimates the financial and economic costs of the 2018 influenza vaccination programme as the base case, as well as the costs of two expansion scenarios where coverage is increased by an absolute 5% and 10% from 2018 coverage rates. The perspective taken is that of the public provider. Currently, TIV is provided as part of the annual influenza vaccination campaign in South Africa. However, there is ongoing global discussion around replacing TIV with the more effective, but more expensive, quadrivalent inactivated influenza vaccine (QIV).^22^, ^23^ In order to inform future discussions by policy makers, we also estimate the costs of the programme under a fourth scenario where TIV is replaced with QIV.^23^

## 2 METHODS

To support decision making and implementation efforts in countries, WHO has developed the ‘Influenza Vaccine Economic Value Chain’ guidance package for influenza vaccination.^16^, ^17^ This package includes a Microsoft Excel‐based planning and costing tool to estimate influenza vaccination costs for the different influenza risk groups—The WHO Seasonal Influenza Immunization Costing Tool (WHO SIICT).^16^, ^17^ Further details on the tool can be found in the Supporting Information.

We adapted the WHO SIICT to estimate the cost of South Africa's seasonal influenza vaccination strategy. Using data obtained from primary and secondary sources in South Africa as well as some pre‐populated tool inputs, we estimated total financial and economic costs of the programme as well as cost per fully immunised individual. Due to the existence of the South African programme, start‐up costs were not included in this analysis. Financial costs are defined as the monetary outlays required to deliver the immunisation programme, while economic costs include, in addition to financial cost, the opportunity costs of using existing resources in the course of delivering the programme.^20^, ^24^ For example, financial costs would include the money spent to procure doses of vaccines, while economic costs would additionally include time spent on the vaccine programme by existing staff.

### 2.1 Primary data collection

We collected data from November 2018 to February 2019, prior to the commencement of the 2019 seasonal influenza vaccination campaign. Thus, all data collected pertain to the 2018 vaccination campaign. As part of this process, we established a steering committee consisting of vaccination programme managers, experts and decision makers to provide advice on the validity of tool inputs and the relevance of the scenarios modelled. The steering committee was also important in identifying sources of data and facilitating the data collection process.

We used a structured questionnaire designed to collect information on resource‐use quantities, based on the cost categories described in the WHO SIICT (Table S1). Data were collected from different levels of the health care system, including the national, provincial, district/municipal and facility levels. We purposively selected Tshwane District within Gauteng Province as this district has a range of urban, peri‐urban and rural facilities that deliver influenza vaccination, in addition to its convenient location for collection of data within study time and resource constraints. The Supporting Information provides a detailed description of types of resource‐use data collected (Table S2) and the designation of the key informants recruited into the study.

### 2.2 Secondary data collection

Data for other cost inputs were extracted from several secondary sources, which can be found in Table S3. Unit price of the vaccine was obtained from the 2018 Master Procurement Catalogue compiled by the Affordable Medicines Division at the NDoH.^25^ Personnel salaries were obtained from the 2018 South African Department of Public Service and Administration (DPSA) salary scales,^26^ 2018 NDoH and Gauteng Department of Health Annual Reports^27^, ^28^ and from the literature.^29^ To estimate travel costs for planning, training and distribution activities, a rate of South African Rands (ZAR) 3.61 per kilometre (km) was applied, in accordance with South African Revenue Services guidance.^30^ Unit prices of consumables (mainly cotton swabs) were obtained from existing published literature.^31^

The pre‐populated volume of each vaccine in centimetres cubed (cm^3^)^32^ was multiplied by the number of vaccines procured to calculate total cold chain volume used by the programme. This total volume was multiplied by the cost per cm^3^ of the cold chain, obtained from the Programme for Appropriate Technology in Health (PATH) vaccine regional distribution centre cost assessment of 2011,^33^ to estimate the cost of cold chain volume used by the programme. This cost was adjusted to 2018 ZAR using the historical exchange rate and the Statistics South Africa Consumer Price Index.^34^, ^35^

To account for wastage, a vaccine wastage rate of 5% was assumed, in line with median wastage rate for vaccines in single‐dose vials in LMIC.^36^, ^37^ However, costs of disposal of used syringes were deemed negligible due to the small size of the influenza vaccination campaign relative to the Expanded Programme for Immunisation (EPI) programme and were therefore excluded from the analysis.

Finally, coverage estimates for 2018 were obtained from the Post Vaccination Evaluation, conducted by the NDoH,^11^ while population estimates of each risk group were obtained from a study on influenza in risk groups in South Africa.^12^

### 2.3 Base‐case analysis

Total cost of the programme was calculated by multiplying the quantities of resources used by their unit cost and aggregating over activity categories. Cost estimates were generalised to the rest of South Africa by applying cost estimates obtained from our purposive sample in Gauteng to the corresponding numbers of provinces, districts and facilities in South Africa. This assumes that seasonal influenza immunisation activities in Gauteng are representative of immunisation activities conducted in all nine provinces in South Africa. Finally, cost per person immunised was estimated by dividing total programme costs by the estimated number of individuals immunised.^11^ Costs were assumed to be constant across the risk groups, as the activities and cost ingredients involved in administering the vaccine at the PHC facility were the same across groups.

### 2.4 Scenario analyses

We modelled two expansion scenarios to assess total costs of expanding current coverage of the influenza vaccination programme in South Africa, first by an absolute increase of 5% and second, by an absolute increase of 10% within each risk group. These scenarios were considered by steering committee members to be realistic scale‐up options for the seasonal influenza vaccination programme in South Africa. The scenarios amounted to procurement of an additional 968,275 and 1,936,550 doses, respectively, reflecting a likely gradual expansion of the programme due to financial constraints. The current coverage rates for each high‐risk group as well as coverage rates for both scenarios are provided in Table 1. In modelling expansion of the programme, not all cost categories and ingredients were expanded proportionately. For example, reporting and training activities are conducted periodically throughout the influenza season, and expansion of the programme is unlikely to influence the amount of time and resources required to conduct these activities. Thus, costs associated with reporting and training were kept constant in each expansion scenario. On the other hand, procurement, immunisation activities, distribution and social mobilisation activities are likely to increase proportionately with an increase in coverage. In order to ensure adequate uptake of the vaccine in the expansion scenarios, we assumed that additional social mobilisation activities would be required and adjusted these costs accordingly in each scenario.

**TABLE 1:** Seasonal influenza vaccine coverage rates for the high‐risk target groups in South Africa, 2018

Target population	Population estimate (in millions)	Doses administered	Current vaccine coverage (%)	Coverage in expansion Scenario 1^a^ (%)	Coverage in expansion Scenario 2^b^ (%)
Adults >65 years	3.015^38^	93,766^11^	3.11	8.11	13.11
Pregnant women	0.925^39^	160,148^11^	17.31	22.31	27.31
Underlying medical conditions	8.995^40^	282,438^11^	3.14	8.14	13.14
HIV infected	6.430^39^	354,324^11^	5.51	10.51	15.51
Total high‐risk populations	19.366	890,676	4.60	9.60	14.60

^a^ Absolute increase in vaccine coverage by 5%.

^b^ Absolute increase in vaccine coverage by 10%.

Furthermore, we assumed that in the expansion scenario, 50% of the additional vaccine recipients would visit health facilities solely for the purpose of being vaccinated against influenza. This assumption was based on the assessment of steering committee members involved in the management and implementation of the seasonal influenza vaccination programme in South Africa. Therefore, under these scenarios, we applied a facility fee to account for the additional burden on the health care system, obtained from the Uniform Patient Fee Schedule of 2018.^41^ This assumption may not hold for pregnant women who would receive the vaccine during antenatal visits even in expansion scenarios. Therefore, a facility fee was not applied to pregnant women in any of the expansion scenarios.

Additional costs introduced in the expansion scenario include costs of expanding cold chain capacity to accommodate additional vaccines. Given that the EPI cold chain system in South Africa is currently operating at full capacity, any additional cold chain capacity required would require capital infrastructure investments. Given space constraints within health facilities, we assumed that capital investments in cold chain would occur only at the district level. Therefore, in both expansion scenarios, we modelled an increase in regional pharmacy cold chain capacity and a corresponding increase in the frequency of vaccine distribution from regional pharmacies to health facilities. Capital costs of procuring additional refrigerators were annualised using a discount rate of 5% and a 10‐year useful life.^42^, ^43^

Finally, we modelled a scenario in which TIV was replaced with the more expensive QIV in the current vaccination programme, using the same coverage rate as for the base‐case scenario. In the absence of a QIV cost per dose, we assumed that the cost of QIV was 150% the cost of TIV.^44^, ^45^

## 3 RESULTS

### 3.1 Financial costs: Base‐case analysis

Estimates of the financial costs of the influenza vaccination programme in 2018 are presented in Table 2. Costs were calculated in ZAR and converted to USD using a rate of ZAR 13.24 to USD 1.00.^34^ In 2018, approximately 900,000 doses of influenza vaccines were available in the public health system, covering approximately 5% of individuals in the risk groups, resulting in a total financial programme cost of approximately ZAR 38.8 million (USD 2.93 million) and a cost per person immunised of ZAR 43.61 (USD 3.29) (Table 2; Panel A). The largest financial cost driver was procurement of the vaccines, contributing approximately 99% of total financial cost (Table 3). Distribution, training and microplanning also contributed to financial cost but to a much lesser degree (Table 3).

**TABLE 2:** Costs of the seasonal influenza vaccination programme as implemented in South Africa in 2018

	Base‐case scenario	5% expansion	10% expansion	Quadrivalent vaccine
Number of doses procured	937,553	1,951,898	2,968,587	937,553
Number of doses administered (Coverage %)	890,676 (4.60)	1,858,951 (9.60)	2,827,226 (14.60)	890,676 (4.60)
Panel A: Total costs				
Total financial costs (ZAR [USD])	38,845,910 [2,934,131]	81,276,367 [6,139,011]	123,706,823 [9,343,892]	57,700,108 [4,358,236]
Total economic costs (ZAR [USD])	104,706,781 [7,908,770]	230,465,618 [17,407,656]	356,224,454 [26,906,541]	12,560,979 [9,332,876]
Financial cost per person immunised (ZAR [USD])	43.61 [3.29]	43.72 [3.30]	43.76 [3.30]	64.78 [4.89]
Economic cost per person immunised (ZAR [USD])	117.56 [8.88]	123.98 [9.36]	126.00 [9.52]	138.73 [10.48]
Panel B: Total costs disaggregated by vaccine procurement cost and service delivery costs				
Total vaccine procurement cost	37,708,396 [2,848,211]	78,702,110 [5,944,571]	119,695,825 [9,040,931]	56,562,594 [4,272,317]
Total (economic) vaccine service delivery cost	66,998,385 [5,060,559]	151,763,507 [11,463,085]	236,528,630 [17,865,610]	66,998,385 [5,060,559]
Vaccine procurement cost per person immunised	42.34 [3.20]	42.34 [3.20]	42.34 [3.20]	63.51 [4.80]
Vaccine service delivery cost per person immunised	75.22 [5.68]	81.64 [6.17]	83.66 [6.32]	75.22 [5.68]

Abbreviations: USD, United States dollars; ZAR, South African rands.

**TABLE 3:** Financial cost drivers for the four study scenarios modelled, South Africa, 2018

Programme activity	Base‐case scenario	5% scale‐up	10% scale‐up	Quadrivalent vaccine
Microplanning	0.003%	0.001%	0.001%	0.002%
Procurement	98.97%	98.72%	98.64%	99.31%
Distribution	0.993%	0.977%	0.972%	0.669%
Training	0.031%	0.015%	0.010%	0.021%
Cold chain expansion (annualised)	0%	0.289%	0.379%	0%

### 3.2 Financial costs: Scenario analyses

Total financial cost increased to ZAR 81.3 million (USD 6.14 million) if current coverage within each risk group was increased by 5% and ZAR 124 million (USD 9.34 million) if increased by 10% (Table 2). Cost per person immunised was estimated at ZAR 43.72 (USD 3.30) and ZAR 43.76 (USD 3.30) in the 5% and 10% expansion scenarios, respectively (Table 2; Panel A). As with the base‐case scenario, procurement costs accounted for the highest proportion of total financial costs in both expansion scenarios (Table 3). Cold chain expansion was included as a financial cost in the expansion scenarios but was not a large cost driver (Table 3).

We observed an increase in financial cost of the programme to ZAR 57.7 million (USD 4.36 million) when TIV was replaced with QIV, at base‐case coverage rates. Similarly, financial cost per person immunised increased to ZAR 64.78 (Table 2).

### 3.3 Economic costs: Base‐case analysis

Estimates of economic costs of the programme are presented in Table 2. When opportunity costs of the programme were considered (such as staff time and existing cold chain infrastructure), total economic cost was estimated at approximately ZAR 105 million (USD 7.91 million) and cost per person immunised at approximately ZAR 117.56 (USD 8.88) (Table 2; Panel A). Economic cost of the programme was largely driven by procurement (37%), immunisation activities (32%) and social mobilisation (11%), as shown in Table 4. Overall, vaccine delivery cost represented approximately 63% of total economic costs, with vaccine procurement costs accounting for the remainder. Vaccine delivery costs and vaccine procurement costs per person immunised were estimated at ZAR 75.22 (USD 5.68) and ZAR 42.34 (USD 3.20), respectively (Table 2; Panel B).

**TABLE 4:** Economic cost drivers for the four study scenarios modelled, South Africa, 2018

Programme activity	Base‐case scenario	5% scale‐up	10% scale‐up	Quadrivalent vaccine
Microplanning	2.09%	0.95%	0.61%	1.77%
Procurement	36.72%	34.81%	34.25%	46.37%
Distribution	0.57%	0.53%	0.52%	0.48%
Training	8.29%	3.77%	2.44%	7.03%
Social mobilisation	11.27%	8.45%	7.62%	9.55%
Immunisation activities	31.62%	44.65%	48.48%	26.80%
Supervision/monitoring activities	9.19%	6.62%	5.86%	7.79%
Current cold chain utilised	0.26%	0.12%	0.08%	0.22%
Cold chain expansion (annualised)	0%	0.10%	0.13%	0%

### 3.4 Economic costs: Scenario analyses

Overall, total economic costs increased to ZAR 230 million (USD 17.4 million) and ZAR 356 million (USD 26.9 million) when the current programme was expanded by 5% and 10%, respectively. Similarly, economic cost per person immunised increased to ZAR 123.98 (USD 9.36) and ZAR 126.00 (USD 9.52) in the 5% and 10% expansion scenarios, respectively (Table 2). In addition to procurement costs, economic costs associated with immunisation activities were observed to increase with each expansion scenario (Table 4). This was largely due to the facility fee applied to account for non‐opportunistic vaccinations in these scenarios. Replacing TIV with QIV also resulted in an increase in total economic costs and cost per person immunised (Table 2; Panel A), with procurement costs accounting for the difference between those scenarios (Table 2; Panel B).

## 4 DISCUSSION

In this study, we estimated the financial and economic costs of the seasonal influenza vaccination programme in South Africa. In addition, we modelled two expansion scenarios to estimate the cost of expanding vaccine coverage in vulnerable individuals and the implications of changing the vaccine administered from TIV to QIV. In 2018, approximately 5% of individuals at risk of developing more severe consequences of influenza were covered by the programme, resulting in a total financial cost of ZAR 38.8 million (USD 2.93 million) and a financial cost per person immunised of ZAR 43.61 (USD 3.29). This represents approximately 2% of the total expenditure on vaccines in South Africa.^46^, ^47^ Given similar vaccine delivery platforms, all costs per person immunised are constant across all risk groups evaluated.

Vaccine procurement was a significant driver of both financial and economic costs. Discussions with pharmaceutical companies around potential cost‐savings associated with larger dose purchases may introduce some economies of scale in the expansion scenarios. However, we observed an increasing contribution of activities relating to the administration of vaccines in the expansion scenarios when opportunity costs were accounted for, leading to increased economic cost per immunised individual. This indicates that economies of scale have limited applicability in this programme and shows the importance of accounting for opportunity costs in a costing study such as this, where economic costs may be a better reflection of the impact of the intervention on the health system than financial costs.

Although cost estimates across settings are generally not comparable due to differences in health system costs, we observed similar patterns in a study estimating the cost of immunising health workers in Albania.^48^ Vaccine procurement cost was the highest financial cost driver of the Albanian programme.^48^ Another costing study applied similar methods in Malawi to prospectively estimate the costs of introducing a maternal influenza immunisation programme, and found microplanning, training and social mobilisation to be the largest financial cost drivers,^49^ contrary to our findings. This is largely due to the assumption that influenza vaccines were donated and were thus considered an economic cost input, rather than a financial one.^49^ This shows the importance of local data, as the costs of an influenza vaccination programme might vary depending on country‐specific contextual factors.

### 4.1 Limitations

We collected data from only one province (out of nine), one district (out of 52) and six facilities (out of approximately 3500). We assumed that the programme in Gauteng Province was representative of the programme across all nine provinces. Although each province operates a facility‐based programme, the number of facilities in each district, the number of patients served at each facility and management of the programme are likely to differ across provinces. The extent to which these variations may have biased our estimated costs is unclear and future studies may expand data collection to a more representative sample of provinces, districts and facilities in South Africa.

## 5 CONCLUSION

This study demonstrates the successful use of the WHO SIICT costing tool to estimate financial and economic costs associated with the current influenza vaccination programme in South Africa. Furthermore, it provides evidence to decision makers wishing to understand the potential costs of both expanding the current influenza vaccination efforts and replacing TIV with QIV. As next steps, we suggest the use of cost estimates reported in this study as integral inputs to (i) a budget impact analysis, which considers potential savings from averted influenza‐associated illnesses and (ii) an assessment of the cost‐effectiveness of QIV compared to TIV.

## AUTHOR CONTRIBUTIONS

**Heather Fraser:** Conceptualization; data curation; formal analysis; investigation; methodology; project administration; software; visualization. **Winfrida Tombe‐Mdewa:** Data curation; formal analysis; investigation; methodology; project administration. **Ciaran Kohli‐Lynch:** Data curation; formal analysis; investigation; methodology; visualization. **Karen Hofman:** Funding acquisition; project administration; resources; supervision. **Stefano Tempia:** Conceptualization; data curation; formal analysis; investigation; methodology; supervision. **Meredith McMorrow:** Data curation; investigation; methodology; supervision; visualization. **Philipp Lambach:** Conceptualization; methodology; project administration; resources; software; supervision. **Wayne Ramkrishna:** Data curation; investigation; supervision. **Cheryl Cohen:** Formal analysis; investigation; methodology; supervision. **Raymond Hutubessy:** Conceptualization; funding acquisition; resources; software; supervision. **Ijeoma Edoka:** Conceptualization; data curation; formal analysis; funding acquisition; investigation; methodology; project administration; supervision; validation; visualization.

## DISCLAIMER

Raymond C. W. Hutubessy and Philipp Lambach work for the World Health Organization. The authors alone are responsible for the views expressed in this publication, and they do not necessarily represent the decisions, policy or views of the World Health Organization.

### PEER REVIEW

The peer review history for this article is available at https://publons.com/publon/10.1111/irv.12987.

## Supporting information




**Table S1.** Programme activity categories in the Seasonal Influenza Immunization Costing Tool in South Africa, 2018
**Table S2.** Cost ingredients obtained through primary data collection, Tshwane District, South Africa, 2018
**Table S3.** Seasonal Influenza Immunization Costing Tool model inputs obtained from secondary sources, South Africa, 2018

## Data Availability

The populated tool used in the study will be made available upon request.
