# Supplementary material for: Costs of seasonal influenza vaccination in South Africa
Source: Influenza Other Respir Viruses. 2022 Mar 30;16(5):873–80. doi: 10.1111/irv.12987 (PMC9343325; doi:10.1111/irv.12987)
Supplement: Supplementary file 1 — Table S1. Programme activity categories in the Seasonal Influenza Immunization Costing Tool in South Africa, 2018 Table S2. Cost ingredients obtained through primary data collection, Tshwane District, South Africa, 2018 Table S3. Seasonal Influenza Immunization Costing Tool model inputs obtained from secondary sources, South Africa, 2018 [file IRV-16-873-s001.docx]

Supplementary materials

Overview of the Influenza Vaccination Programme in South Africa

According to the National Influenza Policy and Strategic Plan, provincial health departments are responsible for implementing the annual influenza vaccination programme in South Africa ^[1]^. Following national guidelines, provincial departments develop comprehensive plans for conducting vaccination. These plans include training of staff, social mobilisation, distribution of vaccines from regional pharmacies to facilities, monitoring and evaluation, management and reporting. Vaccines are procured by provincial health departments under a procurement agreement with a pharmaceutical company who distributes the vaccines from the manufacturer to regional pharmacies. The price paid to the pharmaceutical company includes costs related to freight, insurance, taxes, and the pharmaceutical company also develops and distributes social mobilisation materials (banners, posters, and radio advertisements).

The vaccination campaign typically coincides with the influenza season, running from approximately late March to September each year ^[2]^. TIV is administered free of charge at primary healthcare (PHC) facilities. Administration of the vaccination is opportunistic, with high-risk individuals vaccinated during visits for routine or acute care. No supplementary immunisation activities (SIAs) are conducted and the delivery platform for the vaccination programme is entirely through healthcare facilities ^[1]^. TIV is also available through the private sector in South Africa, funded through health insurance or out of pocket payments; however, this study considered only the public sector programme.

**Details of the WHO SIICT Tool**

The WHO SIICT is a Microsoft Excel based tool that enables researchers and vaccination programme managers to estimate the costs of introducing influenza vaccination for different risk groups and estimate the incremental costs of what is already ongoing in a country.

The WHO SIICT makes use of an ingredient-based costing approach (i.e. the quantities of resources used and their unit cost were collected separately) to estimate programme costs from a public provider perspective and includes cost categories pertaining to only the direct costs borne by the health system in delivering the programme, hence omitting costs borne by individuals and households ^[3]^. These cost categories include microplanning, procurement, distribution, training, social mobilisation, vaccine administration/immunisation activities, supervision and monitoring, cold chain expansion, and ‘other’ immunisation activities (Table S1).

Table S1. Programme activity categories in the Seasonal Influenza Immunization Costing Tool in South Africa, 2018 ^[4]^

| **Programme Activity** | **Description** | **Cost Inputs** |
| --- | --- | --- |
| **Microplanning** | Development of comprehensive operational plans for immunisation activities at the different levels of the health system. Usually done in meetings. | Personnel time  Transport  Per diems and travel allowances  Venue rental |
| **Procurement^†^** | The process of acquiring vaccines, supplies and safety boxes | Cost of single dose of vaccine in a vial, syringes and needles, and safety boxes  Freight  Insurance  Taxes/Subsidies/Customs clearance  Storage |
| **Distribution** | The process of delivering immunisation supplies from regional pharmacies to primary healthcare facilities | Personnel time  Transportation costs |
| **Training** | Any training that needs to be conducted to support the immunisation program | Personnel time  Training materials (compilation and printing costs)  Travelling costs  Allowances  Venue hire |
| **Social mobilisation** | Sensitization activities to inform the community about immunisation activities | Personnel time  Printed material e.g. posters |
| **Supervision and monitoring** | Monitoring and reporting of immunisation activities | Personnel time  Expenses  Travelling costs  Allowances |
| **Immunisation activities** | Activities carried out as part of administering the vaccine: for example time spent injecting individuals and recording doses administered | Personnel time  Consumables |
| **Cold chain expansion** | Calculation of cold chain requirements | Volume of vaccine refrigeration used |

**^†^**In the South African procurement agreement, certain social mobilization activities conducted by the pharmaceutical company are included in the procurement price of the vaccine. For example, cost of information, education and communication (IEC) materials (posters and banners) as well as cost of radio advertisements are included in the vaccine price per dose.

**Roles and designation of the key informants**

At the national level, key informants recruited into the study included a National Communicable Disease Control Manager; a representative of the pharmaceutical company contracted to manage the procurement and distribution of the vaccines on behalf of the NDoH; and a programme manager responsible for health promotion activities at the national level. At the provincial level, the Provincial Communicable Disease Control Co-ordinator was interviewed. At the district level, the Expanded Programme for Immunisation (EPI) programme manager and the regional pharmacy manager were interviewed. At the facility level, facility managers and nurses involved in administering vaccines (or otherwise involved in the programme) were interviewed. In total, six primary healthcare facilities formed the convenience sample in Tshwane District, including two urban, two peri-urban, and two rural facilities.

Table S2. Cost ingredients obtained through primary data collection, Tshwane District, South Africa, 2018

| **Health System Level** | **Category of activities** | **Cost ingredients** | **Resource use** | **Designation of personnel** | **Monthly salary estimates** |
| --- | --- | --- | --- | --- | --- |
| **National level** | Planning | - Hours spent planning for programme roll-out | - 134 hours - 12 hours - 100kms | - Communicable Disease Control Manager - Grade I medical scientist | - ZAR 95 310 (USD 7 199) ^[5]^ |
|  | Training | - Hours spent on capacity building activities | - 100 hours | - Communicable Disease Control Manager | - ZAR 95 310 (USD 7 199) ^[5]^ |
|  | Social mobilisation | - Hours spent on seasonal influenza vaccine health promotion activities | - 7 full days (112 hours total) | - Health Promotion Coordinators at the National level | - ZAR 61 750 (USD 4 664) ^[5]^ |
|  | Supervision, monitoring & reporting | - Hours spent on reviewing provincial reports, reporting to WHO, consulting with NICD - Hours spent on reporting on service delivery activities | - 124 hours - 130 hours | - Communicable Disease Control Manager Grade I medical scientist | - ZAR 95 310 (USD 7 199) - ZAR 32 128 (USD 2 427)   ^[5]^ |
| **Provincial level** | Planning | - Hours spent on budget allocation, initiation of procurement process, reviewing district micro-plans | - 8 hours - 64 hours | - Provincial Communicable Disease Control coordinator - Finance officer, - Supply chain manager | - ZAR 100 416 (USD 7 585) - ZAR 63 427 (USD 4 791) - ZAR 63 427 (USD 4 791) ^[6]^ |
|  | Procurement | - Total cost for procurement of 260 000 vaccines | - ZAR 40.22 per vaccine (USD 3.04) |  |  |
|  | Training | - Hours spent at Provincial training sessions | - 32 hours - 32 hours | - Senior managers - Highly-skilled supervisors | - ZAR 100 416 (USD 7 585) - ZAR 63 427 (USD 4 791) ^[6]^ |
|  | Supervision, monitoring & reporting | - Hours spent compiling end of season report - Hours spent on influenza surveillance | - 8 hours - 52 hours | - Senior manager - Highly-skilled supervisor | - ZAR 100 416 (USD 7 585) - ZAR 63 427 (USD 4 791) ^[6]^ |

Table S2. Continued: Cost ingredients obtained through primary data collection, Tshwane District, South Africa, 2018

| **Health System Level** | **Category of activities** | **Cost ingredients** | **Resource use** | **Designation of personnel** | **Monthly salary estimates** |
| --- | --- | --- | --- | --- | --- |
| **District level** | Planning | - Develop micro-plans for the district - Preparing vaccine stock forms | - 16 hours - 80 hours (5 co-ordinators for 2 days) - 16 hours | - District coordinator - Sub-district coordinators - Regional pharmacy manager | - ZAR 63 427 (USD 4 791) ^[6]^ - ZAR 28 042 (USD 2 118) ^[6]^ - ZAR 84 524 (USD 6 384) ^[7]^ |
|  | Distribution | - Hours spent delivering seasonal influenza vaccines - Distance driven from regional pharmacy to facilities (cost per kilometre in Table S2) | - 32 hours (2 drivers for 2 days) - 500km per driver per day, for 2 days = 2000kms | - Regional pharmacy drivers | - ZAR 17 916 (USD 1 353) ^[6]^ |
|  | Training | - Hours spent at district training - Hours spent on influenza training at sub-district meetings | - 32 hours - 42 hours | - District coordinator - Sub-district coordinators | - ZAR 63 427 (USD 4 791) - ZAR 28 042 (USD 2 118) ^[6]^ |
|  | Social mobilisation | - Hours spent doing health promotion at facilities | - 5 working days (total 200 hours) | - Sub-district Chief Liaison Officers | - ZAR 28 042 (USD 2 118) ^[6]^ |
|  | Cold chain | - Capacity of regional pharmacy cold chain used to store vaccines (volume of packaged vaccines in Table S2) | - Average of 432,646cm^3^  required per district |  |  |
|  | Supervision, monitoring & reporting | - Collecting data from facilities - Collating data collected from facilities into the DHIS - End of season report (to send to Province) - Pharmacy report | - 8 hours - 72 hours - 8 hours | - District coordinator - Sub-district coordinators - Regional pharmacy manager | - ZAR 63 427 (USD 4 791) ^[6]^ - ZAR 28 042 (USD 2 118) ^[6]^ - ZAR 84 524 (USD 6 384) ^[7]^ |

Table S2. Continued: Cost ingredients obtained through primary data collection, Tshwane District, South Africa, 2018

| **Health System Level** | **Category of activities** | **Cost ingredients** | **Resource use** | **Designation of personnel** | **Monthly salary estimates** |
| --- | --- | --- | --- | --- | --- |
| **Facility level** | Planning | - Hours spent attending district planning meetings - Distance travelled to planning meetings (cost per kilometre in Table S2) | - 1.8 hours - 4.3kms | - Facility managers | - ZAR 47 779 (USD 3 609) ^[7]^ |
|  | Immunisation activities | Time spent administering of vaccine and recording of doses administered – measured per person immunised | 3.5 minutes | - Nurses | - ZAR 27 775 (USD 2 098) ^[7]^ |
|  | Cold chain | Volume of cold chain capacity utilised at the facility (volume of packaged vaccines in Table S2) | 5980cm^3^ per facility required |  |  |
|  | Social mobilisation | - Time spent on daily talks with patients - Time spent doing community talks and radio shows | - 8 hours - 53 hours | - 1 Nurse - 1 community health worker | - ZAR 27 775 (USD 2 098) ^[7]^ - ZAR 3 500 (USD 264) ^[8]^ |
|  | Training | - Hours spent attending district and facility in-service training - Distance travelled to district training sessions (cost per kilometre in Table S2) | - 6 hours - 1.3 hours - 65km | - Nurses - Facility managers | - ZAR 27 775 (USD 2 098) - ZAR 47 779 (USD 3 609) ^[7]^ |

Table S3. Seasonal Influenza Immunization Costing Tool model inputs obtained from secondary sources, South Africa, 2018

| **Model Input** | **Value** | **Source** |
| --- | --- | --- |
| **Cold chain: Cost of cold chain capacity** | ZAR 0.0711 (USD 0.00537) per cm^3^ – regional pharmacy storage  ZAR 0.329 (USD 0.0249) per cm^3^ – facility level storage | PATH Vaccine regional distribution center cost assessment 2011 ^[9]^ |
| **Cold chain: Volume of packaged vaccine** | 26.3 cm^3^ | WHO prequalified vaccines: pre-populated model input ^[10]^ |
| **Cold chain: Refrigerator useful life years** | 10 years | Pre-populated model input ^[4]^ |
| **Consumables: cotton swabs** | ZAR 0.82 (USD 0.06) per immunised individual | Previous economic evaluation conducted in South Africa used as reference case ^[11]^ |
| **Vaccine wastage** | 5% | Pre-populated model input, confirmed in the literature ^[4,12]^ |
| **Facility visit fee** | ZAR 103 (USD 7.78) per visit | Uniform Patient Fee Schedule 2018 ^[13]^ |
| **Cost per km travelled** | ZAR 3.61 (USD 0.27) per km | South African Revenue Services guidelines ^[14]^ |
| **Personnel salaries (USD per month)** | Ranging from ZAR 3 500 – ZAR 100 416 (USD 264 - USD 7 585) | National Department of Health Annual Report 2018 ^[5]^  Gauteng Department of Health Annual report 2017/18 ^[6]^  Department of Public Service and Administration (DPSA) Salary Scales ^[7]^  Literature ^[8]^ |

**References:**

1. National Department of Health. National Influenza Policy and Strategic Plan 2017 to 2021 [Internet]. 2017 [cited 2019 Jun 18]. Available from: http://www.health.gov.za/index.php/component/phocadownload/category/339#

2. Biggerstaff M, Cohen C, Reed C, Tempia S, McMorrow ML, Walaza S, et al. A cost-effectiveness analysis of antenatal influenza vaccination among HIV-infected and HIV-uninfected pregnant women in South Africa. Vaccine 2019;37(46):6874–84.

3. Sanders GD, Neumann PJ, Basu A, Brock DW, Feeny D, Krahn M, et al. Recommendations for Conduct, Methodological Practices, and Reporting of Cost-effectiveness Analyses: Second Panel on Cost-Effectiveness in Health and MedicineRecommendations From the Second Panel on Cost-Effectiveness in Health and MedicineRecommendations From the Second Panel on Cost-Effectiveness in Health and Medicine. JAMA 2016;316(10):1093–103.

4. de Boer P, Postma M, Lambach P, Hutubessy R. WHO Seasonal Influenza Immunization Costing Tool. Geneva: World Health Organization; 2018.

5. National Department of Health. Annual Report 2017/2018. 2018.

6. Gauteng Department of Health. Annual Report 2017/2018. 2018.

7. Department of Public Service and Administration. Salary scales, with translation keys, for employees on salary levels 1 to 12 and those employees covered by Occupation Specific Dispensations (OSDs) [Internet]. 2018 [cited 2019 Jun 18]. Available from: http://www.saou.co.za/wp-content/uploads/2016/04/18_1_p_20_06_2018_Appendices_A.pdf

8. Goudge J, de Kadt J, Babalola O, Muteba M, Tseng Y, Malatji H, et al. Household coverage, quality and costs of care provided by community health worker teams and the determining factors: findings from a mixed methods study in South Africa. BMJ Open 2020;10(8):e035578.

9. PATH. Vaccine Regional Distribution Center Cost Assessment. Seattle: PATH; 2011.

10. World Health Organization. WHO Prequalified Vaccines [Internet]. 2016;Available from: https://extranet.who.int/pqweb/vaccines/list-prequalified-vaccines

11. Meyer-Rath G, van Rensburg C, Chiu C, Leuner R, Jamieson L, Cohen S. The per-patient costs of HIV services in South Africa: Systematic review and application in the South African HIV Investment Case. PLOS ONE 2019;14(2):e0210497.

12. Parmar D, Baruwa EM, Zuber P, Kone S. Impact of wastage on single and multi-dose vaccine vials: Implications for introducing pneumococcal vaccines in developing countries. Hum Vaccin 2010;6(3):270–8.

13. National Department of Health. Uniform Patient Fee Schedule [Internet]. 2018 [cited 2019 Aug 15];Available from: http://www.health.gov.za/index.php/shortcodes/2015-03-29-10-42-47/2015-04-30-09-10-23/uniform-patient-fee-schedule/category/448-upfs-2018#

14. South African Revenue Service. SARS: Rates per kilometre [Internet]. 2019;Available from: https://www.sars.gov.za/Tax-Rates/Employers/Pages/Rates-per-kilometer.aspx
